# Effects of different physiotherapy modalities on insomnia and depression in perimenopausal, menopausal, and post-menopausal women: a systematic review

**DOI:** 10.1186/s12905-023-02515-9

**Published:** 2023-07-08

**Authors:** Hagar E. Lialy, Malak A. Mohamed, Latifa A. AbdAllatif, Maria Khalid, Abdulrahman Elhelbawy

**Affiliations:** 1 Faculty of Physical Therapy, Cairo University, Giza, Egypt; 2 Faculty of Medicine, Helwan University, Cairo, Egypt

**Keywords:** Physiotherapy, Insomnia, Depression, Exercise, Reflexology, Walking, Massage, Yoga

## Abstract

**Background:**

Menopause is the time that marks passing 12 months after the last menstruation cycle in women between ages 40–50. Menopausal women often experience depression and insomnia that significantly impact their overall well-being and quality of life. This systematic review aims to determine the effects of different therapeutic physiotherapy modalities on insomnia and depression in perimenopausal, menopausal, and post-menopausal women.

**Methodology:**

After identifying our inclusion/exclusion criteria, we conducted a database search in Ovid Embase, MIDRIS, PubMed, Cochrane, and ScienceOpen, where 4007 papers were identified. By using EndNote software, we excluded duplicates, unrelated, and non-full text papers. Adding more studies from manual search, we finally included 31 papers including 7 physiotherapy modalities: exercise, reflexology, footbath, walking, therapeutic and aromatherapy massage, craniofacial message, and yoga.

**Results:**

Reflexology, yoga, walking and aromatherapy massage showed an overall significant impact on decreasing insomnia and depression in menopausal women. Most of exercise and stretching interventions also showed improvement in sleep quality but inconsistent findings regarding depression. However, insufficient evidence was found regarding the effect of craniofacial massage, footbath, and acupressure on improving sleep quality and depression in menopausal women.

**Conclusion:**

Using non-pharmaceutical interventions such as therapeutic and manual physiotherapy have an overall positive impact on reducing insomnia and depression in menopausal women.

**Supplementary Information:**

The online version contains supplementary material available at 10.1186/s12905-023-02515-9.

## Background

Menopause is the time that marks passing 12 months after the last menstruation cycle. It happens in elderly women between 40 and 50 s with a mean age of 51 [1]. Menopause has three phases: perimenopause, menopause, and postmenopause. Perimenopause phase represents the last few years before menopause when women start to have irregular menstrual cycles and hot flashes. Menopause is about experiencing a full year without a monthly menstrual cycle. In this phase, many menopausal symptoms start to show up. Post-menopause phase demonstrates the rest of women’s life after menopause [2].

The transition to menopause can be a challenging time for women, as it is associated with a range of physical and psychological symptoms that affect most of menopausal women. Depression and insomnia are two common symptoms that women experience during the menopausal transition. These symptoms can negatively affect their health, well-being, and daily activities, leading to a decline in their overall quality of life [3].

Depression is a mood disorder that is characterized by feeling of sadness, loss of interest or pleasure, and a range of other emotional and physical symptoms [4]. Depression in menopausal women happens due to fluctuating and declining of estrogen levels in the body. Studies showed that women without a pre-existing depression history are at a high-risk of getting depression during menopause with a 16% prevalence of new-onset depression and/or anxiety [6].

Furthermore, menopause has a negative impact on sleep quality, causing insomnia. Insomnia is a sleep disorder that is characterized by difficulty falling asleep or staying asleep [4]. A survey of over 12,000 women in a study of women’s health across nations (SWAN) reported that nearly 40% of women have a difficulty with sleeping during menopausal transition [6].

Treatment for menopause symptoms includes Hormonal therapy (HT), non-hormonal medications, or non-pharmacologic therapies. While HT can be effective in alleviating menopausal symptoms, its use is associated with several risks, including an increased risk of breast cancer, stroke, and blood clots [5]. HT is only safe and effective for women without any underlying coronary heart disease or history of breast cancer and for those less than 60 years old [7]. That means the risks of HT in some cases may outweigh the benefits, and alternative treatments should be considered.

In contrast, physiotherapy is a non-hormonal and non-pharmacological intervention that has been suggested to be effective in improving both insomnia and depression in menopausal women. Previous studies assured that physiotherapy has an effective role in dealing with menopausal symptoms with no adverse effects on their health. It increases hormones levels such as serotonin and the serum concentration of the brain-derived neurotrophic factor, thus, reducing depression and improving sleep without any change in oxidative stress [8, 9].

The aim of this study is to provide an updated comprehensive review of the existing literature to identify the most recent and effective physiotherapy modalities for the treatment of insomnia and depression in perimenopausal, menopausal, and post-menopausal women. We aim to fill any gaps in the literature and highlight areas for future research. The findings of this study will be useful for clinicians and researchers in developing effective physiotherapy interventions and providing evidence-based recommendations for women experiencing insomnia and depression during the menopausal transition.

## Methodology

### Search strategy

To find studies addressing the effects of various physiotherapy modalities on perimenopausal, menopausal, and postmenopausal women suffering from insomnia and depression, a systematic search was conducted in the following electronic databases: Cochrane, PubMed, ScienceOpen, Ovid Embase, and MIDRIS. The final search term was developed using combinations of keywords and Boolean operators. (Table 3 in Supplementary files)

### Selection criteria

Based on our PICO (P; Women who were perimenopausal, menopausal, or postmenopausal at the time of the study they participated at) (I; Different physiotherapy modalities) (C; A different type of physiotherapy intervention or no intervention (control group)) (O: Effects on depression and insomnia), we identified our inclusion and exclusion criteria.

We included papers that were published in the last 12 years (2010–2022), have open-accessed full texts, and written in English language only. Any study with non-invasive physiotherapy intervention conducted in any geography and setting was eligible for inclusion.

Our exclusion criteria included invasive physiotherapy modalities such as acupuncture, women with induced menopause (because of surgical removal of ovaries, etc.), menopausal women who use contraceptives or under Hormonal Replacement Therapy (HTR), menopausal women suffering from other pathological conditions such as cancer, cardiac diseases, or thyroid gland disorders, women with primarily depression and insomnia that are not because of menopause, and papers addressing the effects of combined interventions of physiotherapy and pharmacological or hormonal therapy.

### Data extraction

The studies’ IDs, aim, population, protocol, assessment tools, and conclusion were extracted in Table 1.

**Table 1:** Table of characteristics

Study ID and Country	Aim	Population	Protocol	Assessment tools	Conclusion
Valeh 2020 [10] Iran	Comparing the effects of eight weeks of low, moderate, and high intensity TRX training on hot flashes, mood, fat percentage and muscular endurance of postmenopausal women.	40 postmenopausal Women with the mean age 50.4 divided into 4 equal groups: 1) control (2) low-intensity TRX training (3) moderate-intensity TRX training (4) high-intensity TRX training.	8 weeks of Low, moderate, and high-intensity TRX training.	- Brunel questionnaire - Kupperman index questionnaire	Adaptation to regular exercise reduces the symptoms of menopause. In this study, there was no significant difference between using different intensities of training.
Sternfeld 2013 [11] USA	Determining the efficacy of exercise training for alleviating vasomotor and other menopausal symptoms.	248 of pre- and post-menopausal women with mean age 55.8. Intervention group n = 106, Control group n = 142	12 weeks of three individualized cardiovascular conditioning training sessions per week.	- Patient Health Questionnaire-8 and Generalized Anxiety Disorder-7 questionnaire - Insomnia Severity Index	Exercise training may slightly improve subjective sleep quality and symptoms of insomnia and depression.
Moilanen 2012 [12] Finland	Assessing whether aerobic training affects menopausal symptoms in recently postmenopausal sedentary women.	151 menopause women with the mean age 54.5 Intervention group n = 74 Control group n = 77	The 6 months aerobic training. Four times per week, with 50 min of exercise at a time.	-Women’s Health Questionnaires’	Aerobic training for 6 months improve sleep quality among symptomatic menopausal women.
Mansikkamäki2021 [13] Finland	Assessing effects of exercise on sleep quality or nighttime hot flushes among menopausal women	169 menopause women with mean age 54 Intervention n = 83 Control n = 86	six-month unsupervised aerobic training intervention (50 min 4 times per week)	-Women’s Health Questionnaire -Mobile phone questionnaire	Aerobic training may improve sleep quality and reduce hot flushes related to sleep disturbance among midlife women.
Asghari 2016 [14] Iran	Assessing the effect of exercise and nutrition education on quality of life and early menopausal symptoms.	108 pre and menopausal women divided into 4 groups equally: 1.Nutrition 2. Exercise 3. Nutrition plus exercise 4. Control	The aerobic exercises were performed as walking for 12 weeks three times per week for 30–45 min.	- Menopause specific QOL (MENQOL) - Greene menopausal Scale	Nutrition education with aerobic exercise can improve quality of life.
Dąbrowska 2016 [15] Poland	Investigating the influence of a 12-week training program on the quality of life in menopausal-aged women living in a rural area.	80 women with mean age 51 ± 3.82 Intervention n = 40 Control n = 40	12 Weeks training, 3 times a week. Each session is 60-minute included warming-up, walking, stretching, strengthening exercises with an elastic band, and cooling down exercises.	-Short Form Health Survey (SF36)	The training was significantly correlated with a positive change in vitality and mental health.
Aibar-Almazan 2019 [16] Spain	Analyzing the effects of Pilates-based exercise program has on sleep quality, anxiety, depression, and fatigue in postmenopausal women.	107 postmenopausal women with the mean age 68.18 ± 8.35 Intervention group n = 55 Control group n = 52	12 weeks Pilates system training. Each Pilates training session was divided into three parts: warm-up (10 min), main Pilates training activity (35 min), and cool-down (15 min).	-Pittsburgh Sleep Quality Index (PSQI) -The Hospital Anxiety and Depression Scale (HADS)	Pilates exercise intervention has beneficial effects on sleep quality, anxiety, depression, and fatigue.
Kai 2016 [17] Japan	Assessing the effects of a 3 weeks stretching program on the menopausal and symptoms in middle-aged Japanese women	40 women aged between 40 to 61 stretching group n = 20 Control group n = 20	3 weeks of stretching program with a daily 10 min	-Simplified Menopausal Index. -Self-Rating Depression Scale.	10 min of stretching exercise before sleep participate in Reducing Depression symptoms in menopausal women.
Takahashi 2019 [18] Japan	Assessing the effects of physical activity for depression biomarkers in postmenopausal women	38 of postmenopausal women mean age of (70.2 ± 3.9) Control group n = 19 Intervention group n = 19	The participants do daily physical activity for 8 weeks	-Geriatric Depression Scale (GDS). -Quality of life scale (QOL)	Daily physical activity decrease depression in postmenopausal women.
Hu 2017 [19] China	Assessing the effect of walking on menopausal symptoms in community-dwelling postmenopausal Chinese women.	80 Women between the ages of 45 to 65 Control group n = 40 Intervention group n = 40	Patients walks 3 times per week for 16 weeks.	-The 21-item Beck Depression Inventory (BDI) - Menopause Rating Scale - ANOVAs	Walking had a significant effect on Reducing Depression and menopause symptoms.
Bernarda 2014 [20] France	Assessing whether walking can reduce depression incidence in inactive post-menopausal women without depression.	121 women with the mean age 57 ± 75. Intervention group = 61 Control group = 60	Patients perform type of walking called ACTi ‘march program, three times a week, 40 minutes per session, for six-months.	- Beck Depression Inventory (BDI) -PAQE -ANCOVA - partial eta-squared	Walking reduces incidence of depression symptoms in inactive post-menopausal women without depression.
Noh 2020 [21] Korea	Investigating effects of 12-week SaBang-DolGi walking exercise program on the physical and mental health of menopausal women.	40 women aged between 50 to 65. Intervention group = 21 Control group = 19	12-week SaBang-DolGi walking, three sessions / week, 60 min a day.	-simple mental health test II Korea. -Symptom-Checklist-90-Revision (SCL-95-R).	SaBang-DolGi walking exercise program reduces Depression and promoting the level of mental and physical health of menopausal women who face stress and depression.
Tadayon 2016 [22] Iran	Evaluating the effects of walking with a pedometer on the sleep quality of postmenopausal Iranian women.	112 women Intervention group = 56 Control group = 56	Walking on pedometer each day for 12 weeks, 500 steps per week.	- PSQI	Walking with a pedometer has an efficient role to improve the quality of sleep among postmenopausal women.
Aghamoham-madi 2020 [23] Iran	Determining the effect of footbath on sleep disturbance in postmenopausal women.	100 women with mean age 53.42 ± 1.84 Intervention group = 50 Control group = 50	Patients put their feet into warm water with a temperature of 41 to 42 °C, for 20 min in a plastic container with a depth of 10 cm one hour before the usual sleeping time for 6 weeks.	- PSQI -Greene scale -ANCOVA	Footbath intervention improved sleep quality in menopausal women.
Aydin 2021 [24] Turkey	Determining the effect of reflexology on insomnia and fatigue.	72 postmenopausal women Intervention group = 36 Control group = 36	A 6-week, 12-session foot reflexology program; 2 sessions/week; 30 min./session;15 min. for each foot	-PSQI -Fatigue Severity Scale (FSS)	Reflexology program increased sleep quality and decreased fatigue.
Asltoghiri 2011 [25] Iran	Determining the effects of reflexology on hot flashes and sleep quality in menopausal women	100 menopausal women ranging from 40 to 65 years. Intervention group = 53 Control group = 47	A 21-day reflexology program, 15-min session daily vs. non-specific foot massage	-PSQI	Reflexology is an effective non-hormonal approach for treatment of sleep disorder.
Mahdavipour 2019 [26] Iran	Determining the effects of foot reflexology on depression during menopause.	90 menopausal women. Intervention = 45 Control groups = 45	15 min of 3-stage foot reflexology on each foot for a total of 30 min in evenings, twice a week for six weeks.	-Beck Depression Inventory-second edition (BDI-II)	Foot reflexology technique can be effective for reducing women’s depression during menopause
Portella 2020 [27] Brazil	To investigate the effects of yoga on insomnia and other Menopausal symptoms.	47 women between the ages of 40 to 55 Intervention group = 18 Control group = 15	45 min/day during for 8 weeks.	-Kupperman Menopausal Index (KMI) -Insomnia Severity Index (ISI) -Pittsburgh Sleep Questionnaire (PSQ) -Berlin Questionnaire	Yoga contributes to improvement of Menopausal symptoms and a treatment option of insomnia during menopausal transition
Swain 2021 [28] India	Assessing the effects of yoga on menopausal symptoms, and changes in hormonal levels among menopausal women.	80 participants aged 40 to 50. Intervention group = 40 Control group = 40	-Sudarshan Kriya Yoga (SKY) sessions for a 1-year, three sessions weekly for 45–60 min of each session. -Control group practice brisk walking daily during the early morning for 30–40 min.	-Menopausal quality of life assessment (MENQOL) -Hormonal level (ELISA)	1 year of SKY could be one of the preferred non hormonal therapy for improving quality of life in menopause.
Afonso 2011 [29] Sao Paola	Evaluating the effect of yoga practice on the physical, mental health and climacteric symptoms of postmenopausal women with a diagnosis of insomnia.	44 postmenopausal women between the ages of 50 to 65 year divided into 3 groups: Control group = 15 Passive stretch group = 14 Yoga = 15	The two groups have two sessions per Week, each session is one hour	-Beck Anxiety Inventory -Beck Depression Inventory -Kupperman Menopausal Index -Insomnia Severity Index -MENQOL, -Inventory of Stress Symptoms for Adults.	Yoga is effective in decreasing insomnia and improving quality of life.
Joshi 2010 [30] India	Evaluating the effect of yoga practicing on menopausal women mental health.	180 women Intervention group = 90 Control group = 90	Practicing yoga one hour daily for three months.	Menopause Rating Scale (MRS)	Yoga is effective in reducing menopause symptoms.
Xi Lu 2020 [31] China	Evaluating the effect of the information support method combined with yoga on the depression, anxiety, and sleep quality of Menopausal women.	106 women between the age of 45 and 55. Intervention group = 52 control group = 54	Yoga exercise for 60 min each time, three times a week for 24 weeks.	-The Kupperman Index (KMI) -Self-rating Depression Scale -Self-rating Anxiety Scale -PSQI	Support method combined with yoga exercise can decrease depression and anxiety of menopausal women, improving their sleep quality.
Oliveira 2012 [32] Brazil	Evaluating effect of therapeutic massage on insomnia and climacteric symptoms in post-menopause.	44 postmenopausal women. Intervention group = 15 Passive movement = 14 Control group = 15	4-month therapeutic massage (TM) and passive movement (PM) program.	-MENQOL -Beck Anxiety Inventory - KMI -BDI -polysomnography (PSG) - Insomnia Severity Index	Massage exhibited improved quality of life and sleep quality and a decrease in depressive symptoms.
Darsareh 2012 [33] Iran	Determining the effect of aromatherapy and placebo massage on menopausal Symptoms.	90 postmenopausal women Aromatherapy massage (n = 30) placebo massage (n = 30) control group (n = 30)	30-minute aromatherapy massage or therapeutic message with odorless soft paraffin oil treatment sessions at a room temperature of 24-C to 26-C, twice a week for 4 weeks	Menopause Rating Scale (MRS)	Both massage and aromatherapy massage were effective in reducing menopausal symptoms.
Taavoni 2013 [34] Iran	Determining the effect of aromatherapy massage on psychological symptoms during menopause				Massage with or without aromatherapy improves psychological symptoms.
Esp´ı-Lopez 2020 [35] Spain	Investigating the effects of therapeutic craniofacial massage on quality of life, mental health and menopausal symptoms and body image	50 postmenopausal women. craniofacial massage group (n = 25) control group (n = 25)	one session a week of 30-minute craniofacial massage for 3 weeks with a follow-up of one month	-MRS -Mental Health scale of the SF-36 quality of life questionnaire -Body Satisfaction and Global Self Perception questionnaire (QSCPGSe)	Craniofacial massage is effective in improving mental health and quality of life.
Susanti 2022 [37] Indonesia	investigating the effects of yoga on menopausal symptoms and sleep quality across menopause statuses.	208 menopausal women aged 45–60 years. Intervention group = 104 Control group = 140	Three 75-min sessions per week for 20 weeks. Each session started with a warmup, after which the participants practiced 12 yoga postures.	-the Depression, Anxiety, and Stress Scale -Multidimensional Scale of Perceived -Social Support Menopause Rating Scale -Pittsburgh Sleep Quality Index.	Yoga effectively decreased menopausal symptoms.
Abedian 2015 [38] Iran	Evaluating the effectiveness of acupressure on sleep quality of postmenopausal women.	120 menopausal women aged 41–65 years. Acupressure group = 37 Sham acupressure group = 36 Control group = 32	- The participants in the acupressure group were asked to massage the effective pressure points and the participants in the sham acupressure group were asked to massage the sham pressure points as a self-care method at home. The intervention time was limited to 10 min. It was done 1 to 2 h before sleeping, each night (Except Fridays) by circular massage covering 1 cm diameter. - The control group only received the weekly control of blood pressure and speech communication about health questions.	-Pittsburgh Sleep Quality Index.	Acupressure can be used as a complementary treatment to relieve sleep disorders in menopausal women.
Eman 2017 [39] Egypt	Comparing foot reflexology and aerobic exercise in decreasing depression.	40 postmenopausal women aged 45–55 Aerobic exercise = 20 Reflexology = 20	-Group A treated by aerobic exercise 40 min, three times/ week for four weeks -Group B was treated with foot reflexology for four weeks.	Beck Depression Inventory (BDI)	Both groups were effective in reducing depression, however aerobic exercise were more effective than reflexology.
Lotfipur Rafsanjani 2015 [40] Iran	Determining the effect of aromatherapy massage on depression in menopausal women	118 postmenopausal women suffering from depression (score of 14 or more on BDI) randomly divided intro: aromatherapy massage = 40 massage therapy = 38 control group = 40	Aromatherapy massage group received a 30-min session/week for 8 weeks. Treatment included massaging with geranium oil (2%) in almond oil. Massage therapy group received a 30 min massage per week only with sweet almond oil for 8 weeks. The control group received the usual control of their general condition.	Beck Depression Inventory (BDI)	Both aromatherapy massage and massage therapy had a more positive impact on reducing depression than control group, but aromatherapy was better.
Márcia P. Jorge 2016 [41]	Studying the psychophysiological effects of Hatha Yoga regular practice in post-menopausal women.	117 volunteers initially included, excluding 29 dropouts, the number of participants assessed were Yoga = 40 Exercise = 29 Control = 19	12 weeks of yoga practice for one group and physical exercise. 75 min of supervised yoga or exercises twice a week, for 12 weeks	-Menopause rating scale -Lipp stress symptoms inventory -BDI -Brief world health organization quality of life questionnaire -State/Trait anxiety inventories -Biochemical parameters	Yoga reflects a significant effect on reducing menopause symptoms, depression, stress level, improving quality of life, and preventing cortisol increase in post-menopausal women.

### Risk of bias assessment

The risk of bias of the included studies was assessed using Cochrane risk of bias tool for randomized controlled trials [36] (Table 2).

**Table 2:** Risk of bias

Study ID	Random sequence generation	Allocation concealment	Blinding of participants and personnel	Incomplete outcome data	Selective reporting	Other bias
Valeh 2020 [10]	Unclear	Unclear	Unclear	Low	Low	Unclear
Sternfeld 2013 [11]	Low	Low	Unclear	Low	Low	Low
Moilanen 2012 [12]	Low	Low	Unclear	low	High	Low
Mansikkamäki 2021 [14]	Low	Low	Unclear	Low	Low	Unclear
Asghari2016 [14]	Low	Low	Unclear	Low	Low	Unclear
Dąbrowska 2016 [15]	Low	High	Unclear	Low	Low	Unclear
Aibar-Almazan 2019 [16]	Low	High	Low	Low	Low	Unclear
Kai 2016 [17]	Low	High	Unclear	Low	High	Unclear
Takahashi 2019 [18]	Unclear	High	High	Low	Low	Unclear
Hu 2017 [19]	Unclear	High	High	Low	low	unclear
Bernarda 2014 [20]	Low	High	Low	Low	Low	Low
Noh 2020 [21]	Unclear	High	Unclear	Low	Low	Unclear
Tadayon 2016 [22]	Low	Low	Unclear	Low	Unclear	Unclear
Aghamoham-madi 2020 [23] low		Low	Unclear	Low	Low	Low
Aydin 2021 [24]	Low	Low	Unclear	Low	Low	High
Asltoghiri 2011 [25]	Unclear	Unclear	Unclear	High	High	Unclear
Mahdavipour 2019 [26]	Low	Low	Low	Low	Low	High
Portella 2020 [27]	Low	High	Unclear	Low	High	High
Swain 2021 [28]	High	Unclear	Unclear	Low	Low	High
Afonso 2011 [29]	Unclear	Unclear	Unclear	High	High	High
Joshi 2010 [30]	Low	Low	Unclear	Low	Low	Unclear
Xi Lu 2020 [31]	Unclear	Unclear	Unclear	Low	Low	High
Oliveira 2012 [32]	Unclear	Unclear	Unclear	Low	Low	Unclear
Darsareh 2012 [33]	Low	Unclear	Unclear	Low	High	Low
Taavoni 2013 [34]	High	High	High	Low	Low	Low
Esp´ı-Lopez 2020 [35]	Low	Low	Unclear	Low	Low	Low
Susanti 2022 [37]	Low	Low	unclear	Low	Low	Low
Abedian 2015 [38]	Low	Low	Low	Low	Low	Low
Eman 2017 [39]	Low	Unclear	Unclear	Low	Low	Low
Lotfipur Rafsanjani 2015 [40]	Unclear	Low	Unclear	Low	Low	Unclear
Márcia P. Jorge 2016 [41]	High	Low	Low	Low	Low	Low

## Results

A total of 4007 studies were retrieved from the 5 databases: PubMed (n = 1065), Cochrane (n = 2693), ScienceOpen (n = 199), Ovid Embase and MIDRIS (n = 49). We used EndNote [42] to identify and delete duplicates (n = 818), and 3189 studies remained.

**Fig. 1:**
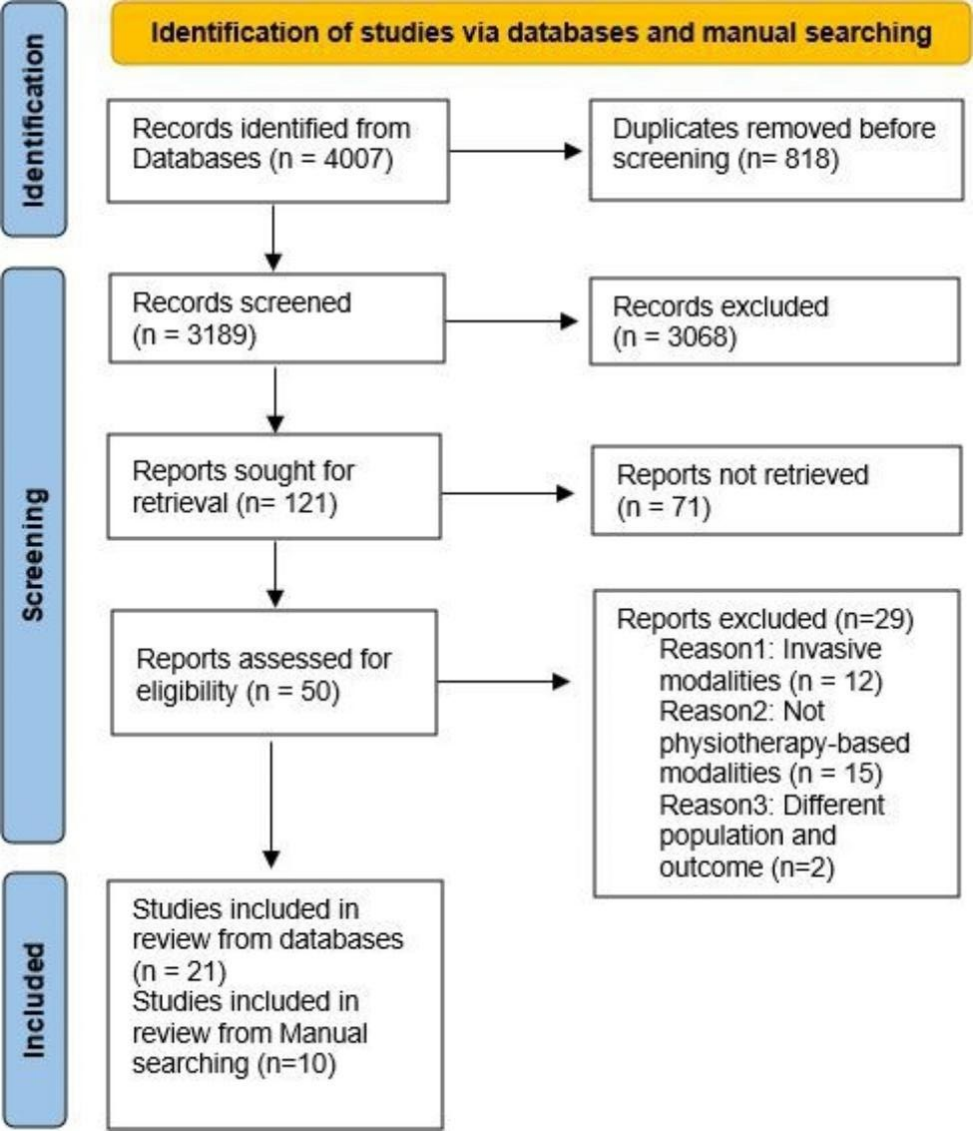
PRISMA Flowchart

According to our inclusion/exclusion criteria, we excluded 3068 papers. After deletion of non-full text studies (n = 71), the full-text papers assessed for eligibility were 50. During the full-text screening, more irrelevant studies of invasive and non-physiotherapy-based modalities or different population or outcomes were excluded. Only 21 studies from the databases and 10 studies from manual search were included, illustrated in PRISMA flowchart [43] (Fig. 1). The included physiotherapy modalities are exercise (n = 9), reflexology (n = 4), walking (n = 4), massage (n = 5), yoga (n = 7), footbath (n = 1), and acupressure (n = 1) with a total number of 2,705 menopausal women in both intervention and control group.

Due to the heterogeneity of the data, all studies were analyzed using qualitative analysis.

### Exercise

#### Aerobic exercise

Sternfeld et al. [11] showed a slight improvement in menopausal symptoms including depression and insomnia in both exercise and usual activity groups, with exercise being more effective. Another study [13] (Mansikkamäki et al.) reported a significant improvement in sleep quality in the aerobic exercise group compared to the control group (p = 0.043).

Moilanen et al. [12] also showed that the perceived disturbance in mood swings (P < 0.001) decreased more in the aerobic exercise group than in the control group. However, there was no significant difference between the two groups regarding depressive mood.

#### Aerobic exercise with nutrition education

One Study [14] reported that mixing aerobic exercise with nutrition education was more effective than using exercise only for improving depression scores ((pre; 28.2, post; 14.2) vs. (pre; 27.2, post; 17.7), respectively).

#### Total body resistance exercise (TRX)

Valeh et al. [10] showed a significant improvement regarding negative mood in the training groups compared to the control group: scores of control group are pre = 12.36 ± 0.95 and post = 12.06 ± 1.2, low-intensity resistance exercise group are pre = 12.31 ± 0.97 and post = 6.99 ± 0.79, moderate-intensity group are pre = 12.31 ± 0.95 and post = 6.98 ± 0.88, and high-intensity group are pre = 11.84 ± 1.19 and post = 6.9 ± 0.62, in which a lower score means less depressive symptoms.

#### Stretching, strengthening, warming up, and cooling down exercises

Dąbrowska et al. [15]used different types of exercise (warming-up exercises, walking, stretching, strengthening exercises with an elastic band, and cooling-down exercises) to improve menopausal mental health. The mental health scores showed the effectiveness of physical activity in improving mental health in menopausal women compared to no intervention. Kai et al. [17] also found that depression scores decreased by 20% from the baseline in the stretching group. Nonetheless, 5 of 12 depressed women in the stretching group recovered to get back to normal after intervention [41.7%], compared to the recovery of only 2 from 13 participants in the control group [15.4%].

However, Takahashi et al. [18] showed no significant difference in depression scores between the active group and the control group. However, the study reported that increasing physical activity reduces depression by improving depression biomarkers. Daily exercise significantly increased brain-derived neurotrophic factor (BDNF) and serum serotonin concentration, which reduce depression symptoms with a p < 0.05.

#### Pilates system

*Aibar-Almazan et al.* [16] showed that Pilates reduced menopausal depression significantly, as the post-intervention scores of depression scale (HADS) (the higher, the more depressive) were 3.98 ± 2.93 for Pilates and 6.81 ± 3.6 for the control group. Furthermore, pre-intervention vs. post-intervention scores of insomnia (PSQI) (the higher, the poorer sleep quality) were 8.56 ± 4.98 vs. 7.16 ± 4.9 in the Pilates group and 7.10 ± 4.42 vs. 8.38 ± 4.28 in the control group. This shows that the sleep quality also improved after the Pilates program.

### Walking

Walking has a significant impact on improving overall mental health, and reducing menopausal symptoms, including sleep problems, and depression.

Two studies [19], [20] reported that walking reduced depression scores and the incidence of depression in menopausal women. Noh et al. [21] also reported that the SaBang-DolGi walking program had positive effects on the physical and mental health of the intervention group. The mean difference in depression scores before and after the intervention in the walking group is -4.81, while it is -1.05 in the control group.

Regarding insomnia, Tadayon et al. [22] reported a reduction in sleep disorders after 12-week walking with a pedometer program, whereas there was no change in the control group.

### Footbath

One Study found [23] a significant effect of footbath on improving sleep quality in the intervention group compared to the control group; the length of sleep in the footbath group increased from [456 min.] to [498 min.] with MD of + 38 min, while it did not significantly change in the control group (421 to 425 min., MD = + 3 min.) The PSQI sub-scores of sleep duration, efficiency, quality, latency, dysfunction, disturbances, and use of sleep medications in the footbath group significantly improved with a PSQI total score MD of -4.67, indicating better scores and fewer sleep problems.

### Reflexology

The studies indicate that reflexology decreases sleep problems and depressive symptoms.

*Asltoghiri et al.* [25] reported that reflexology enhances sleep quality better than non-specific foot massage, as 41.5% of women in the reflexology group reported no sleep disorders after the intervention, compared to non-specific foot massage, which contributed to normal sleep for only 19.1% of the group participants. Furthermore, *Aydın et al.* [24] showed that reflexology is effective in reducing PSQI scores and sleep dysfunction in postmenopausal women in intervention group (pre; 11.4 ± 2.0, post; 6.1 ± 1.5) compared to control group (pre; 10.4 ± 3.1, post; 13.0 ± 2.8). Another study [26] also found that a program of a 3-stage foot reflexology twice a week for 6 weeks reduced depression scores in menopausal women from 26.97 ± 4.47 to 22.55 ± 5.18 after the end of intervention, and to 21.20 ± 5.74 two months after the intervention.

*Eman et al.* [39] compared the effects of reflexology and aerobic exercise on depressive symptoms. The study concluded that both were effective in reducing depression, however, aerobic exercise were more effective in decreasing the mean depression scores (pre; 20, post; 10) than reflexology (pre; 21, post; 14).

### Yoga

The seven studies reported a significant improvement in insomnia and depression in yoga groups compared to control groups.

*Portella et al.* [27] showed that women who had yoga meditation plus sleep hygiene (M + SH) had less sleep problems and depression than the sleep hygiene only (SH) group. *Swain et al.* [28] also showed that women who regularly practiced Sudarshan Kriya Yoga (SKY) experienced 13% fewer psychosocial problems compared to the brisk walking group, which had increased psychological symptoms by 25%. Another study [29] showed that the yoga caused improvements in the menopausal symptoms including a significant reduction in depression and insomnia, while passive-stretching had no significant impact on depression. Furthermore, *Joshi et al.* [30] reported that at the end of the study, yoga group had significantly reduced depression scores (pre; 9.37 ± 7.28, post; 4.36 ± 4.8) compared to control group (pre; 9.37 ± 7.28, post; 9.2 ± 6.72).

*Xi Lu et al.* [31] showed that the sleep quality of the yoga group significantly improved compared with pre-intervention and control group. *Susanti et al.* [37] also found that yoga contributed positively to improving sleep quality in intervention group (pre; 5.51 ± 5.13, post; 1.77 ± 2.73) compared to control group (pre; 5.93 ± 5.07, post; 8.51 ± 6.49).

### Massage

#### Therapeutic massage

*One study* [32] suggests that therapeutic massage has the greatest impact on improving sleep quality and reducing depression among passive movement and no intervention groups.

#### Aromatherapy and placebo massage

All included studies of aromatherapy massage reported its great impact on reducing depression and sleep problems in menopausal women.

*Darsareh et al.* [33] used the Menopause Rating scale (MRS) to examine the effects of aromatherapy and placebo massage vs. no intervention on reducing severity of menopausal symptoms including sleep disorders and depressive mood. The study reported that aromatherapy massage reduces menopausal symptoms more than massage without aromatherapy, with a MD of -5.96 between them. However, both aromatherapy massage and placebo massage had positive impact on reducing menopausal insomnia and depression compared to the control group, which remained the same. *Tavoni et al.* [34] also showed that aromatherapy massage (MD = − 3.49) caused a significant reduction of depression scores from baseline more than massage therapy (MD=-1.20) and no intervention (MD=-0.379) groups. One more study found that aromatherapy massage caused the most significant reduction in the participants’ depression scores with a MD of -0.51 from baseline scores, followed by massage therapy with a MD of − 0.20, while control group scores remained the same.

#### Craniofacial massage

One study [35] showed that craniofacial massage improves mental health in menopausal women, as MRS scores changed in a positive manner at the end of treatment and continued to improve in the follow-up period.

### Acupressure

*Abedian 2015* [38] reported the effectiveness of self-applied acupressure in reducing sleep problems in postmenopausal women, as sleep quality improved in the acupressure group by 41%, compared to sham acupressure group (17%), and control group (2%).

## Discussion

The aim of this systematic review was to investigate the effects of various physiotherapy modalities, including exercise, yoga, walking, footbath, reflexology, and massage, on insomnia and depressive symptoms in menopausal women.

In terms of insomnia or sleep quality, our findings revealed a limited number of studies for Pilates system, walking, footbath, aromatherapy, and acupressure [16], [22], [33], [42], [38], [23]. However, reflexology and yoga were found to have a positive impact on insomnia, improving sleep quality and reducing sleep disorders, as supported by 8 studies [11], [14], [24], [25], [27], [29], [31], [37]. Among these modalities, yoga emerged as the most common intervention for menopausal women experiencing insomnia in the included studies.

Regarding mental health, specifically depression and mood swings, we found a varying number of studies for each modality. We identified studies for stretching, strengthening, warming up, and cooling down exercises, walking, and aromatherapy [15], [17], [18], [19], [20], [21], [33], [34], [40] and only two studies each for aerobic exercise and reflexology [11], [15], [26], [39]. Similar to the findings related to insomnia, yoga was the most frequently tested intervention for menopausal women with depressive symptoms in the 5 included studies [27], [28], [29], [30], [31]. However, insufficient evidence was available for the effect of TRX, Pilates, and craniofacial massage [10], [16], [35] on depression or insomnia.

Our review indicated that aerobic exercise and TRX had a significant impact on improving menopausal symptoms, including decreasing mood swings in the intervention group. Furthermore, when combined with nutrition education, aerobic exercise demonstrated a positive effect on enhancing the overall quality of life during menopause. The Pilates system showed promise in reducing depression and anxiety in menopausal women, as suggested by one study. Physical activity programs involving warming-up exercises, stretching, strengthening exercises with an elastic band, and cooling-down exercises were associated with a positive impact on overall quality of life. Additionally, reflexology studies indicated a decrease in hot flashes among women, which in turn contributed to reduced fatigue and decreased depression symptoms. Massage, encompassing therapeutic, placebo, aromatherapy, and craniofacial techniques, demonstrated improvements in menopausal quality of life by reducing anxiety and depression in postmenopausal women. Aromatherapy and placebo massage were found to be particularly effective in reducing irritability, mental exhaustion, anxiety, and depression symptoms in menopausal women. Notably, walking intervention studies indicated that adhering to a daily one-hour walking regimen significantly improved depression symptoms and menopausal symptoms in menopausal and post-menopausal women.

A systematic review conducted by Kalra et al. in 2022 [46], which included 27 studies, assessed the effects of various types of physical activities and exercises (such as resistance training, aerobics, walking, Pilates, and aquatic exercises) on physiological and psychological parameters. Their findings align with ours, as they reported a strong association between these activities and a reduced risk of depression, improved mental health, increased BDNF levels, reduced memory impairment, enhanced quality of life (QOL), improved fitness, and other physiological parameters. Similarly, the results of a study by Nguyen et al. in 2020 [47] demonstrated that yoga significantly improves psychological and physical quality of life scores. Their analysis of other types of exercises also indicated positive effects on overall, social, sexual, vasomotor, and menopause-specific QOL scores in women experiencing menopausal symptoms.

Regarding physiotherapy modalities, our focus is primarily centered on exercise, yoga, walking and general physical activity due to their appropriateness and popularity within Egypt, as compared to other physiotherapy modalities. The results clearly indicate a positive impact of exercise and physical activity on improving mental health, mood swings, depression, and sleep quality, consequently enhancing the overall quality of life. However, it is important to note that while physical activity is a crucial aspect that positively influenced both physical and mental well-being in the participants, there are multiple factors that may have been contributed to improved mental health such as social and private life and thus sleep quality.

The underlying causes of physical and mental health complaints in perimenopausal women are multifactorial, involving a complex interplay between physiological and psychosocial factors. Physiological factors commonly observed include autonomic nervous system dysregulation, including alterations in sympathetic tone and/or parasympathetic inhibition, as well as decreased estrogen levels [17].

A previous review conducted by Sternfeld [11] suggests that exercise has a positive effect on mental health by influencing various biological mechanisms. These mechanisms include increased release of neurotransmitters, heightened parasympathetic activation, and distraction from stressful stimuli. As a result, exercise contributes to better quality of life and can alleviate vasomotor symptoms (VMS) experienced during menopause.

In a study by Takahashi et al. [18], several plausible mechanisms were proposed to explain the impact of exercise on mental health and depression biomarkers. The research found that increased daily physical activity significantly raised the concentrations of brain-derived neurotrophic factor (BDNF) in postmenopausal women. BDNF plays a crucial role in reducing depression symptoms among older adults and is also associated with memory impairment. Therefore, increasing physical activity becomes important not only as a form of exercise but also for elevating BDNF levels and positively affecting mental well-being.

Furthermore, physical activity can help combat depression by boosting serotonin concentration. Aging has been linked to impaired brain serotonin transmission, which can contribute to the development of depressive symptoms. Long-term engagement in physical activity can enhance serotonin levels in the brain, thus improving mental health and reducing the risk of depressive disorders.

Studies have shown that regular exercise training not only improves mental health and depression but also positively impacts oxidative stress status. Physical activity increases antioxidant capacity, which in turn reduces oxidative stress and thus depression [44] as elevated oxidative stress has been associated with the development of depression [45].

Encouraging individuals to track their step count can lead to increased physical activity, particularly moderate to vigorous physical activity (MVPA). This tracking method has proven to be a key factor in promoting and maintaining mental health, reducing depression, and improving health-related quality of life.

Considering the complex nature of menopausal symptoms, it is essential to incorporate regular exercise and physical activity into interventions. The frequency of exercise sessions, with at least three sessions per week, has been shown to positively influence mood changes and psychological symptoms. Another form of exercise, stretching, is particularly beneficial for middle-aged women as it improves muscle flexibility and alleviates somatic symptoms such as joint pain and shoulder stiffness.

In a review conducted by Nguyen et al. [41], several mechanisms were proposed to explain the positive effect of exercise on vasomotor symptoms. These mechanisms include increased vagal tone, the influence of stress hormones and parasympathetic activation, and thermoregulatory center activity. These findings suggest that exercise may effectively improve vasomotor symptoms in menopausal women.

In light of the findings mentioned above, our recommendation is to encourage menopausal women to increase their physical activity levels to improve their overall quality of life. Women whose physical activity levels remained stable or decreased during menopause experienced worse quality of life. Increased endorphin levels resulting from exercise positively influence mental health, and engaging in exercise groups can have a positive impact on social functioning.

Quality of life is closely linked to sleep quality, and menopausal women often suffer from insomnia, which significantly affects their daily lives. The relationship between sleep and depressive symptoms has been well-documented, with difficulty falling asleep being strongly associated with subsequent depression.

Stretching exercises performed before bedtime have been shown to decrease sleep latency and contribute to better sleep quality, thereby positively affecting menopausal and depressive symptoms. Acute stretching also suppresses sympathetic nervous activity and increases parasympathetic activity, promoting better sleep. Daily stretching routines over a 28-day period can yield chronic effects on the autonomic nervous system.

In conclusion, our paper aims to provide practical and cost-effective solutions to improve the quality of life for menopausal women. By incorporating exercise and physical activity into daily routines, women can experience positive effects on mental health.

Regarding the time period and number of sessions per week for exercise interventions, several studies [10], [14], [15], [18] included in this review examined the effect of physical activity on the quality of life (QoL) in women with mood changes or depression. These studies observed that engaging in daily physical activity for at least 12 weeks or three sessions per week for 6 months can have a positive impact on mood changes, depression, and physical health in menopausal women. Additionally, incorporating a stretching routine at least five days per week, particularly before bedtime, may improve sleep quality and have positive effects on menopausal and depressive symptoms (Ngamprasertwong et al., 2017).

The stretching program holds particular benefits for busy women, considering the increasing number of working middle-aged women globally. Women are reported to have a twofold higher risk of depression compared to men, and work-related stress and other environmental factors can exacerbate menopausal and depressive symptoms. Therefore, addressing menopausal and depressive symptoms is crucial for promoting health among middle-aged working women. Despite the mental and physical benefits of exercise, women in menopause often engage in low levels of physical activity due to perceived time constraints. Encouraging adherence to a stretching program is important for working women with limited time availability. Another effective method to promote physical activity among busy middle-aged working women is to incorporate physical activity during work by tracking and aiming to achieve a minimum of 10,000 steps per day. This can contribute to maintaining mental health, reducing depression and anxiety, and improving self-esteem. Employers can also support employees by allowing rest times during work, which can positively impact both mental health and work quality [18] (Takahashi et al., 2019).

Furthermore, women who perform domestic housekeeping activities such as cleaning, cooking, and laundry engage in daily physical activity. These activities can be classified as low physical activity (LPA) or moderate to vigorous physical activity (MVPA) based on the nature of the tasks. Both LPA and MVPA have been found to have a positive effect on depression, with MVPA being significantly associated with reduced anxiety symptoms [18] (Takahashi et al., 2019).

Incorporating nutrition education along with a 12-week program of moderate-intensity aerobic exercises has shown additional benefits in improving early menopausal symptoms and QoL. This combination has been found to reduce hot flashes, develop a positive attitude toward exercise, and enhance the overall well-being of women aged 40–60 years (Huang et al., 2011; Su et al., 2014).

Moreover, inactive women aged 40–62 who participated in a 12-week yoga program experienced improvements in their quality of life, including reduced hot flushes, better sleep quality, and fewer depressive symptoms. This intervention may be particularly suitable for women with a high socioeconomic status (Orr et al., 2010).

These findings highlight the importance of exercise, stretching, and physical activity in improving the QoL and overall well-being of menopausal women, providing various options that can be tailored to individual preferences and time constraints. By incorporating these interventions into daily routines, women can experience positive effects on their mental and physical health, leading to a better quality of life.

For menopausal women who have time during the day, engaging in physical activities such as walking, aerobic exercises, and line dancing (12 weeks of physical activity or three sessions per week for 6 months) along with regular stretching before bedtime, combined with a program of nutrition education, can significantly improve sleep quality and depressive symptoms. However, for women with limited time or work commitments, focusing on stretching before bedtime alone can still be beneficial compared to a 12-week exercise program. Additionally, increasing physical activity during work or daily housekeeping tasks, such as cumulative logging of step count and aiming for at least 10,000 steps per day, as well as engaging in activities like cleaning and cooking, can contribute to improving menopausal symptoms and overall quality of life. These strategies are particularly relevant for women with insufficient time or demanding work schedules.

It is important to note that physical activity can be of moderate-to-vigorous intensity or light intensity, both of which can alleviate depressive symptoms [17]. Therefore, women can choose the intensity level that suits their preferences and circumstances, with a reminder that engaging in moderate-to-vigorous physical activity or a combination of intensities may yield greater benefits compared to light intensity alone.

Overall, these steps aim to improve the overall physical and mental well-being, enhance the quality of life, and promote a positive attitude toward menopausal symptoms among women in the menopausal age group. Implementing these strategies for a minimum of 12 weeks can initiate long-term lifestyle changes [14], [15]. Furthermore, these approaches offer time and cost-saving advantages, making them suitable for individuals in low and moderate socioeconomic areas. By providing education and promoting positive attitudes, individuals can experience a reduction in menopausal symptoms. Increasing knowledge levels about menopause is associated with decreased symptom frequency, while maintaining a positive attitude helps mitigate the impact of menopausal symptoms [14].

### Strengths and limitations

Our systematic review is a comprehensive search for several types of physical activity and exercises such as yoga, therapeutic and aromatherapy massage, reflexology, aerobic exercise, nutrition education, walking, stretching and others with different tests and parameters. All included studies are randomized controlled trials which are the most accurate type of studies.

However, this systematic review has some limitations. All included studies measured mental health and sleep problems using self-reported methods. Furthermore, the limited number of studies in some modalities may be considered a limitation that may restrict the ability to draw definitive conclusions. The last limitation is the language restriction.

## Conclusion

Physiotherapy is a safe, non-invasive non-pharmacological intervention for improving sleep quality and depression during menopause. Exercise including aerobic exercise, stretching, strengthening, warming up, and cooling down exercises, yoga, walking, reflexology, and massage may reduce insomnia and depression in perimenopausal, menopausal, and post-menopausal women. Moreover, adding aromatherapy to the therapeutic massage found to be more effective than massage without aromatherapy.

However, more evidence is needed regarding the effects of Pilates, footbath, craniofacial massage, acupressure, and adding nutrition education to exercise on sleep quality and depression. There are also insufficient findings on the effects of walking on reducing insomnia. Thus, we recommend conducting more studies to support the findings of these modalities’ impact on sleep and depression in menopausal women.

### Electronic supplementary material

Below is the link to the electronic supplementary material.

Additional File 1: Search term

## Data Availability

All datasets generated or analyzed during the current study are available in the published article.

## Abbreviations

TXR: Total resistance exercise
