# Correction to: Effects of different physiotherapy modalities on insomnia and depression in perimenopausal, menopausal, and postmenopausal women: a systematic review

**DOI:** 10.1186/s12905-023-02573-z

**Published:** 2023-08-09

**Authors:** Hagar E. Lialy, Malak A. Mohamed, Latifa A. AbdAllatif, Maria Khalid, Abdulrahman Elhelbawy

**Affiliations:** 1 Faculty of Physical Therapy, Cairo University, Giza, Egypt; 2 Faculty of Medicine, Helwan University, Cairo, Egypt

**Correction: BMC Women’s Health (2023) 23:1**

10.1186/s12905-023-02515-9

Following publication of the original article [1], in this article a text has been inserted to the “authors’ contributions” section and the same will read as follow:

**Authors’ contributions**

All authors (H.E.L, M.A.M, L.A.A, M.K, and A.E) contributed to research steps including methodology, qualitative analysis, and proof-reading. Authors H.E.L, M.A.M, L.A.A, M.K, and A.E worked on writing the manuscript. M.A.M and H.E.L contributed to paper reviewing, and H.E.L did the paper drafting.

The original article has been corrected.
