# Supplementary material for: Effects of different physiotherapy modalities on insomnia and depression in perimenopausal, menopausal, and post-menopausal women: a systematic review
Source: BMC Womens Health. 2023 Jul 8;23:363. doi: 10.1186/s12905-023-02515-9 (PMC10329343; doi:10.1186/s12905-023-02515-9)
Supplement: Supplementary file 1 — Additional File 1: Search term [file 12905_2023_2515_MOESM1_ESM.docx]

| Table 3 \| Search term |
| --- |
| ((Clinical Trial[Publication Type] Physiotherapy OR Exercise OR Pilates OR Yoga OR Stretching OR Acupuncture OR Electroacupuncture OR Nonpharmacological Interventions OR Non-drug Interventions OR Non-hormonal Therapy OR Natural Interventions OR Aerobic training OR Aerobic Exercise OR Walking OR Stretching OR Therapeutic Massage OR Aromatherapy OR Reflexology OR Cognitive Behavioural Therapy OR Vibrotherapy OR -Chromotherapy OR Melotherapy OR Aeroionotherapy OR AND (clinicaltrial[Filter])) AND (Clinical Trial[Publication Type] Insomnia OR Sleep disorders OR Sleep quality OR Anxiety OR Fatigue OR Depression OR Depressive symptoms OR Lack of sleep OR Mood OR psychological symptoms AND (clinicaltrial[Filter]))) AND (Clinical Trial[Publication Type] Menopause OR Menopausal OR Perimenopause OR Perimenopausal OR Postmenopause OR Postmenopausal OR Climacteric OR hot flushes AND (clinicaltrial[Filter])). |
